# Terrestrial radiotherapy alters microhardness and surface micromorphology of dental restorative materials: an *in vitro* study

**DOI:** 10.3389/froh.2025.1658558

**Published:** 2025-09-25

**Authors:** Apoorva Walia, Laxmish Mallya, Athiyamaan MS, Dilson Lobo, Ramya Shenoy, Annapoorna Shenoy

**Affiliations:** 1 Department of Conservative Dentistry and Endodontics, Manipal College of Dental Sciences Mangalore, Manipal Academy of Higher Education, Manipal, India; 2 Department of Radiation Oncology, Kasturba Medical College Mangalore, Manipal Academy of Higher Education, Manipal, India; 3 Department of Public Health Dentistry, Manipal College of Dental Sciences Mangalore, Manipal Academy of Higher Education, Manipal, India

**Keywords:** ionizing radiation, microhardness, micromorphology, bulk-fill composite, SEM analysis, head and neck cancer, radiation-induced dental effects, good health and well-being

## Abstract

**Background:**

Therapeutic radiotherapy, commonly used in the treatment of head and neck cancers, may alter the mechanical and surface properties of restorative dental materials. Understanding these changes is essential for ensuring the long-term success of restorations in oncology patients.

**Methods:**

An *in vitro* study was conducted on 90 disc-shaped specimens (*n* = 30 each) of three restorative materials: 3M™ Filtek™ Bulk Fill, Charisma Topaz One, and Cention N. Samples were subjected to two radiation protocols: (i) 70 Gy in 35 fractions (2 Gy/day), and (ii) 45 Gy in 5 fractions (9 Gy/day). Vickers microhardness testing and scanning electron microscopy (SEM) were performed 48 h post-irradiation.

**Results:**

Filtek™ Bulk Fill exhibited the highest pre-radiation hardness (83.1 ± 2.3 HV), followed by Charisma Topaz One (74.5 ± 2.8 HV) and Cention N (69.8 ± 2.1 HV). After exposure to 70 Gy, a statistically significant reduction in microhardness was observed across all materials (*p* < 0.05), with Bulk Fill remaining the least affected (74.3 ± 2.1 HV). SEM images confirmed surface degradation in all groups, with varying degrees of filler particle exposure.

**Conclusions:**

Ionizing radiation alters both microhardness and surface morphology of restorative materials, with bulk-fill composites demonstrating greater resilience. These findings warrant further investigation *in vivo* to understand long-term clinical implications.

## Introduction

Oral cancer represents one of the most frequently encountered malignancies in dental practice, predominantly affecting anatomical sites in the head and neck region. Its management is guided by factors such as tumour location, staging, histopathological differentiation, and overall patient health status (1). Radiotherapy, either alone or in combination with surgery, is a cornerstone treatment modality. It operates through high-energy ionizing radiation, which targets tumor cells while minimizing collateral damage to surrounding healthy tissues (2).

However, ionizing radiation can interact not only with biological tissues but also with restorative dental materials, potentially altering their physicochemical properties (3). This is of particular concern in patients undergoing cervicofacial radiation, where restorative interventions often become more complex. These patients may experience compromised adhesion of restorative materials, leading to restoration failure and increased risk of secondary caries (4, 5). Prior studies have highlighted reduced bond strength and changes in microhardness of enamel and dentin post-irradiation (6), yet limited evidence exists comparing how modern restorative materials—such as bulk-fill composites, resin-modified glass ionomers (RMGIs), and alkasite-based materials—respond to therapeutic doses of radiation (7).

In restorative dentistry, material longevity and biocompatibility are paramount. A key mechanical property that influences the durability of dental restorations is surface microhardness, which reflects a material's resistance to plastic deformation and wear (8). While traditional amalgam restorations have demonstrated long-term clinical success, their use has declined due to aesthetic limitations and concerns about mercury content. Consequently, contemporary restorative practice increasingly favors tooth-colored materials such as resin composites and glass ionomer cements (9).

Among these, newer classes of materials—such as bulk-fill composites, nano-hybrid composites, and alkasite-based restoratives—have been introduced to enhance aesthetics, simplify clinical protocols, and improve mechanical resilience. Bulk-fill composites, for example, allow for incremental layering and exhibit lower polymerization stress, while maintaining adequate depth of cure (10). Charisma Topaz, a nano-hybrid composite, offers enhanced strength and wear resistance owing to its TCD-matrix structure (11). Cention N, an alkasite-based material, features bioactive fillers that release calcium, fluoride, and hydroxide ions, promoting remineralization under acidic conditions (12).

Despite these advancements, limited data exist on how ionizing radiation—common in cancer therapy—affects the surface properties and structural integrity of such materials. Preliminary studies suggest radiation may degrade the resin matrix, disrupt filler-matrix bonding, and reduce surface hardness, thus potentially shortening clinical lifespan (7, 13).

While conventional composites have been widely used in daily practice, new formulations such as nano-hybrid composites and alkasites offer improvements in filler technology, polymer matrix resilience, and ion release. However, their durability under radiation exposure has not been sufficiently validated. A clearer understanding of material degradation, especially at the microstructural level, is needed to guide clinicians in material selection for patients undergoing head and neck radiation therapy (8).

This study aims to evaluate the effect of therapeutic ionizing radiation on the microhardness and micromorphology of three widely used restorative materials: 3M™ Filtek™ Bulk Fill (bulk-fill composite), Charisma Topaz One (nano-hybrid composite), and Cention N (alkasite). Scanning electron microscopy (SEM) and Vickers microhardness testing were employed to assess surface-level changes following two clinically relevant radiation protocols.

We hypothesized that exposure to therapeutic radiation would lead to a significant decrease in surface microhardness and visible morphological degradation of all tested restorative materials, with differences depending on their formulation and filler content.

## Materials and methods

### Study setting and ethics

This *in vitro* study was conducted at the Department of Conservative Dentistry and Endodontics, Manipal College of Dental Sciences, Mangalore, and the Department of Radiation Oncology, Kasturba Medical College, Mangalore. Ethical approval was secured from the Institutional Ethics Committee, Manipal College of Dental Sciences, Mangalore (22059). All protocols followed institutional safety guidelines.

### Sample preparation

Three commercially available restorative materials were used:

- 3M™ Filtek™ Bulk Fill Posterior Restorative (3M ESPE, USA) – a packable bulk-fill composite based on Bis-GMA, UDMA resin matrix with zirconia/silica fillers (∼76.5 wt%).
- Charisma Topaz One (Kulzer GmbH, Germany) – a nano-hybrid composite based on TCD-matrix technology with micro- and nano-fillers.
- Cention N (Ivoclar Vivadent, Liechtenstein) – an alkasite-based restorative containing alkaline glass fillers and a UDMA matrix designed for ion release.

Thirty disc-shaped specimens per material (*n* = 90 total) were fabricated using custom acrylic molds (6 mm diameter, 3 mm thickness), following the protocol described by de Amorim et al. (7). After incremental placement and curing, the specimens were finished using a 600-grit silicon carbide paper under water cooling for 30 s, standardized by a single operator.

### Grouping

Specimens were randomly divided into nine groups (*n* = 10 per group) based on material type and radiation exposure protocol (Table 1). The sample size of *n* = 10 per group was determined based on prior *in vitro* studies assessing radiation effects on restorative materials, providing >80% statistical power (*α* = 0.05) to detect a minimum difference of 5 HV in microhardness (Table 1).

**Table 1:** Sample groups according to the radiation dose and protocol.

Group	Material type	Radiation dose	Protocol description
G1	Cention N (Ivoclar)	0 Gy	Control – No irradiation
G2	Cention N (Ivoclar)	70 Gy	Protocol A: (2 Gy/day, 5 days/week for 7 weeks)
G3	Cention N (Ivoclar)	45 Gy	Protocol B: Fractionated protocol (5 × 9 Gy)
G4	Charisma Topaz One (Kulzer)	0 Gy	Control – No irradiation
G5	Charisma Topaz One (Kulzer)	70 Gy	Protocol A: (2 Gy/day, 5 days/week for 7 weeks)
G6	Charisma Topaz One (Kulzer)	45 Gy	Protocol B: Fractionated protocol (5 × 9 Gy)
G7	Bulk Fill (3M™ Filtek™)	0 Gy	Control – No irradiation
G8	Bulk Fill (3M™ Filtek™)	70 Gy	Protocol A: (2 Gy/day, 5 days/week for 7 weeks)
G9	Bulk Fill (3M™ Filtek™)	45 Gy	Protocol B: Fractionated protocol (5 × 9 Gy)

### Radiation protocol

Radiation was administered using an ELEKTA Compact Linear Accelerator (Elekta AB, Sweden) with a 6 MV photon beam. The source-to-surface distance (SSD) was maintained at 100 cm, and the field size was set at 10 cm × 10 cm.

Two clinically relevant protocols were used:

- Protocol A: 70 Gy delivered in 35 fractions (2 Gy/day, 5 days/week for 7 weeks).
- Protocol B: 45 Gy delivered in 5 fractions (9 Gy/day for 5 consecutive days).

These regimens were selected to simulate standard and hypofractionated clinical exposures, respectively, in head and neck cancer patients (14, 15).

Specimens were immersed in artificial saliva in airtight containers during irradiation. The artificial saliva consisted of 1.5 mmol/L CaCl₂, 0.9 mmol/L KH₂PO₄, 150 mmol/L KCl, 0.05 μg/ml NaF, and 0.1 mol/L Tris buffer, adjusted to pH 7.0, and was freshly prepared each week to maintain chemical stability. Testing was conducted 48 h after radiation exposure.

### Microhardness testing

Microhardness was evaluated using the Vickers Hardness Test (Wilson Hardness Tester, Buehler Inc., USA) (16). A 100 g load was applied for 15 s using a diamond indenter. Each specimen received three indentations, spaced at least 1 mm apart, on the flat surface.

Indentation diagonals were measured using an optical microscope (×40 magnification), and the mean Vickers Hardness Number (VHN) was calculated. The device was calibrated prior to testing using a reference block.

### Scanning electron microscopy (SEM)

Post-microhardness samples were fixed in 2.5% glutaraldehyde, dehydrated in an ascending ethanol series (70%, 90%, 95%, and 100%), and dried using critical point drying. The specimens were sputter-coated with 20 nm gold and mounted on aluminium stubs.

SEM imaging was performed using a JEOL JSM−6380 SEM at an operating voltage of 15 kV, with magnifications ranging from 500× to 2000× to assess surface micromorphology.

### Statistical analysis

Data were analyzed using SPSS version 25.0 (IBM Corp., USA). Microhardness values were assessed using two-way ANOVA followed by Tukey's *post hoc* test. Normality of data was checked using the Shapiro–Wilk test, and Levene's test was used for homogeneity of variances. Statistical significance was set at *p* < 0.05.

## Results

### Microhardness analysis

The mean Vickers Hardness Number (VHN) and standard deviations for each restorative material at baseline and after radiation exposure are summarized in Tables 2–4.

**Table 2:** The mean and standard deviation values of vickers hardness before radiation exposure (microhardness pre radiation).

Restorative material	*N*	Mean	Std. deviation	Minimum	Maximum
Cention *N*	10	52.041	2.78531	46.02	53.92
Charisma topaz one	10	65.342	4.56012	59.53	66.24
3M Bulkfil	10	78.643	5.94264	69.21	82.46

Mean ± SD of VHN values before irradiation.

VHN, Vickers hardness number; SD, standard deviation; Gy, Gray.

**Table 3:** The mean and standard deviation values of vickers hardness post radiation protocol 1 (microhardness after radiation protocol 1).

Restorative material	*N*	Mean	Std. deviation	Minimum	Maximum
Cention *N*	10	49.234	3.90453	45.23	51.34
Charisma topaz one	10	61.325	4.15645	54.34	62.87
3M Bulkfil	10	75.328	5.10403	63.24	78.45

Mean ± SD post 70 Gy.

VHN, Vickers hardness number; SD, standard deviation; Gy, Gray.

**Table 4:** The mean and standard deviation values of vickers hardness post radiation protocol 2- (microhardness after radiation protocol 2).

Restorative material	*N*	Mean	Std. deviation	Minimum	Maximum
Cention *N*	10	48.065	3.45901	44.26	52.76
Charisma topaz one	10	62.054	4.23521	53.28	63.27
3M Bulkfil	10	75.124	5.23415	62.67	79.54

Mean ± SD post 45 Gy.

VHN, Vickers hardness number; SD, standard deviation; Gy, Gray.

At baseline (pre-irradiation), Filtek™ Bulk Fill demonstrated the highest surface microhardness (83.1 ± 2.3 HV), followed by Charisma Topaz One (74.5 ± 2.8 HV) and Cention N (69.8 ± 2.1 HV).

Following Protocol A (70 Gy/35 fractions), all materials showed a statistically significant reduction in VHN. Filtek Bulk Fill retained higher values (74.3 ± 2.1 HV) compared to Charisma Topaz (66.0 ± 2.4 HV) and Cention N (60.9 ± 2.5 HV).

After Protocol B (45 Gy/5 fractions), the reduction in microhardness was similar but less pronounced (e.g., Filtek: 76.2 ± 2.0 HV, Charisma: 69.1 ± 2.2 HV, Cention: 63.8 ± 1.9 HV).

The percentage decrease in VHN ranged from 6.9% to 13.5%, depending on the material and radiation protocol.

Two-way ANOVA showed a significant interaction between material type and radiation exposure (*p* < 0.001). Tukey's *post hoc* test revealed significant differences between groups exposed to 70 Gy and controls (*p* < 0.01), but no statistically significant difference between the 70 Gy and 45 Gy protocols (*p* = 0.23).

### Scanning electron microscopy (SEM) observations

**Figure 1:**
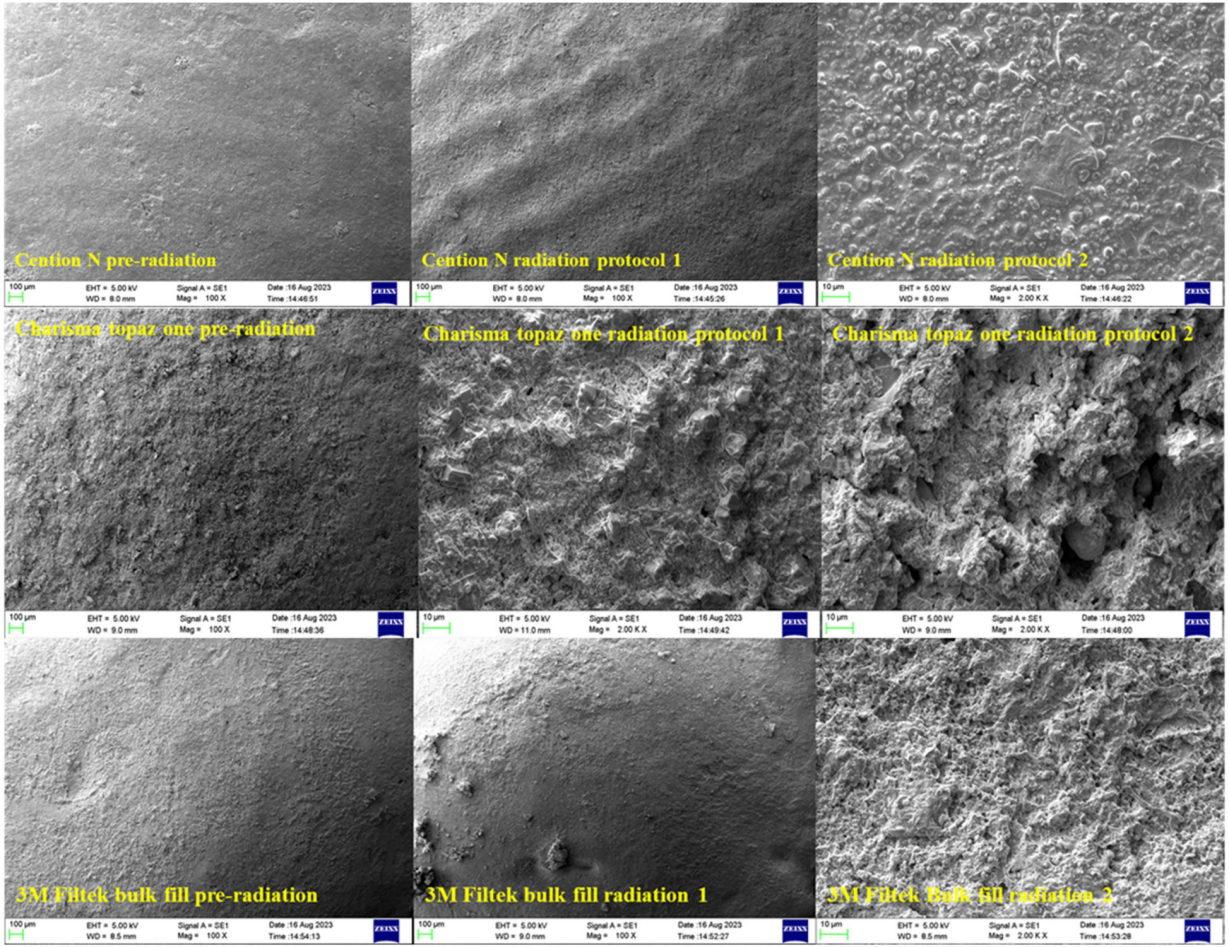
Scanning electron microscope analysis images of Cention N, Charisma topaz one and 3M bulk fill pre and post-irradiation. SEM images of restorative materials pre- and post-radiation (magnifications: ×1,000 and ×2,000).

Representative SEM micrographs are presented in Figure 1.

Filtek™ Bulk Fill specimens showed minor surface degradation with shallow grooves and localized filler particle exposure. The matrix remained mostly intact post-radiation.

Charisma Topaz One exhibited moderate matrix erosion and clustered filler exposure, especially after Protocol A.

Cention N presented the most irregular surface, with noticeable voids, surface cracking, and disrupted filler-matrix continuity.

Across all irradiated groups, filler particle visibility increased, suggesting resin matrix loss and filler dislodgement. No quantitative measurement of surface roughness was performed; however, qualitative grading suggested mild to moderate surface deterioration depending on the radiation dose and material.

## Discussion

This study evaluated the effects of ionizing radiation on the surface microhardness and micromorphology of three contemporary restorative materials. Across all groups, exposure to both standard and hypofractionated radiation protocols led to a measurable reduction in Vickers hardness values and notable changes in surface morphology. These findings confirm that restorative materials can undergo physical degradation under clinically relevant radiotherapy conditions (7). The observed reduction in microhardness post-irradiation may be attributed to chain scission within the resin matrix, oxidative degradation of polymer networks, and debonding at the filler–matrix interface due to differential thermal expansion. Water uptake in irradiated specimens could further plasticize the matrix, contributing to the reduction in hardness (6, 14, 17).

Beyond natural tooth structures, ionizing radiation has also been reported to affect the mechanical and morphological properties of restorative materials. Prior studies have noted degradation in composite resin integrity and changes in their surface topography following therapeutic radiation exposure (7, 18). Our results are consistent with earlier reports showing that ionizing radiation can reduce the mechanical strength of resin-based materials and alter surface texture. Much of this research has been conducted using animal models or outdated materials. The present study sought to address this gap by evaluating the impact of clinically relevant radiation doses on three widely used modern restorative materials: 3M™ Filtek™ Bulk Fill, Charisma Topaz One, and Cention N.

The findings from this study clearly reject the null hypothesis, as exposure to ionizing radiation produced measurable changes in both surface microhardness and micromorphology of the tested materials. Filtek™ Bulk Fill demonstrated the least reduction in hardness values post-irradiation. This may be attributed to its higher filler loading, deeper polymerization capability, and the resilience of its Bis-GMA/UDMA-based resin matrix (10).

While both radiation protocols resulted in a reduction in hardness values, the difference between Protocol A (70 Gy in 35 fractions) and Protocol B (45 Gy in 5 fractions) was not statistically significant (*p* > 0.05). This suggests that material degradation may depend more on total radiation dose than dose fractionation. Nevertheless, subtle microstructural changes observed under SEM imaging support the hypothesis that the radiation-induced damage is material-specific and likely influenced by compositional factors, such as filler size, resin matrix type, and polymerization efficiency.

Charisma Topaz One and Cention N showed more pronounced degradation, with the latter exhibiting surface voids and disrupted filler-matrix integrity on SEM. This is least in Filtek™ Bulk Fill. The detachment of larger filler particles in bulk-fill resins can be explained by the breakdown of the filler–resin interface, likely exacerbated by polymer matrix embrittlement under radiation (19, 20). Interestingly, the smaller and more uniformly distributed fillers in Charisma Topaz appeared to resist matrix detachment better, suggesting that nano-hybrid composites may offer improved resilience in irradiated environments.

The number of exposed filler particles may be correlated with the Vickers hardness values observed, as more exposed fillers often indicate breakdown of the surrounding matrix and thus reduced resistance to indentation (21). Despite this, all three materials demonstrated acceptable levels of mechanical performance post-irradiation, reinforcing their potential use in restorative procedures for oncology patients.

A notable strength of this study is the use of artificial saliva during the irradiation process, which better simulates the oral environment compared to the distilled water used in earlier studies (13). This approach likely improved the external validity of the findings.

However, certain limitations should be acknowledged. This study is not without limitations. As an ex vivo model, it lacks several oral environment variables, including Salivary enzyme interactions, Temperature fluctuations and thermal cycling and Mechanical fatigue due to mastication. Also, only two radiation protocols were tested, which may not capture the full variability encountered clinically *in vivo*, dose distribution can vary based on tumor site, tissue heterogeneity, and beam angulation.

Additionally, as SEM analysis in this study was qualitative, future research should incorporate quantitative profilometry or atomic force microscopy for surface roughness assessment. Nanoindentation testing and polymer crosslink density analysis could further elucidate micromechanical changes. Studies comparing fractionated and single-dose regimens are warranted to determine the clinical relevance of laboratory findings.

### Clinical relevance

Despite these limitations, our findings have important implications for clinicians treating head and neck cancer patients. Restoration margins are particularly vulnerable in irradiated environments due to decreased bonding and higher risk of secondary caries. Selecting materials that are more resistant to radiation-induced degradation—such as bulk-fill composites—could improve restoration longevity and reduce the need for post-treatment replacements or repairs.

These results also underscore the importance of interdisciplinary planning between oncologists and dental professionals to manage restorative care before, during, and after radiotherapy.

## Conclusion

This ex vivo study demonstrated that exposure to therapeutic doses of ionizing radiation significantly affects the microhardness and surface morphology of restorative dental materials. All three tested materials—Filtek™ Bulk Fill, Charisma Topaz One, and Cention N—underwent measurable degradation, with the bulk-fill composite exhibiting greater resistance to these changes.

The findings suggest that material composition, particularly filler type and resin matrix characteristics, influences the extent of radiation-induced alterations. While these results contribute to our understanding of restorative material behavior in irradiated environments, further *in vivo* studies are needed to confirm long-term clinical relevance and performance under real-world oral conditions.

## Data Availability

The raw data supporting the conclusions of this article will be made available by the authors, without undue reservation.
